# Construction and validation of a three-dimensional finite element model of degenerative scoliosis

**DOI:** 10.1186/s13018-015-0334-1

**Published:** 2015-12-24

**Authors:** Jie Zheng, Yonghong Yang, Shuliang Lou, Dongsheng Zhang, Shenghui Liao

**Affiliations:** Department of Orthopaedics, 117 Hospital of PLA, Hangzhou, 310013 China; Central South University, Changsha, 40012 China

**Keywords:** Degenerative, Scoliosis, Finite element analysis, Validation

## Abstract

**Background:**

With the aging of the population, degenerative scoliosis (DS) incidence rate is increasing. In recent years, increasing research on this topic has been carried out, yet biomechanical research on the subject is seldom seen and in vitro biomechanical model of DS nearly cannot be available. The objective of this study was to develop and validate a complete three-dimensional finite element model of DS in order to build the digital platform for further biomechanical study.

**Methods:**

A 55-year-old female DS patient (Suer Pan, ID number was P141986) was selected for this study. This study was performed in accordance with the ethical standards of Declaration of Helsinki and its amendments and was approved by the local ethics committee (117 hospital of PLA ethics committee). Spiral computed tomography (CT) scanning was conducted on the patient’s lumbar spine from the T12 to S1. CT images were then imported into a finite element modeling system. A three-dimensional solid model was then formed from segmentation of the CT scan. The three-dimensional model of each vertebra was then meshed, and material properties were assigned to each element according to the pathological characteristics of DS. Loads and boundary conditions were then applied in such a manner as to simulate in vitro biomechanical experiments conducted on lumbar segments. The results of the model were then compared with experimental results in order to validate the model.

**Results:**

An integral three-dimensional finite element model of DS was built successfully, consisting of 113,682 solid elements, 686 cable elements, 33,329 shell elements, 4968 target elements, 4968 contact elements, totaling 157,635 elements, and 197,374 nodes. The model accurately described the physical features of DS and was geometrically similar to the object of study. The results of analysis with the finite element model agreed closely with in vitro experiments, validating the accuracy of the model.

**Conclusions:**

The three-dimensional finite element model of DS built in this study is clear, reliable, and effective for further biomechanical simulation study of DS.

## Introduction

Degenerative scoliosis (DS) is the constitutional alignment of the spinal column caused by degeneration of the intervertebral disc and facet joints after skeletal maturation in which the coronal Cobb angle is greater than 10° [1, 2]. The disease is usually seen in the lumbar or lower thoracic spine of patients above 50 years of age, so it is often called “scoliosis of the elderly”. With the aging of the population, its incidence rate is increasing. In recent years, increasing research on this topic has been carried out, yet biomechanical research on the subject is seldom seen. With the advancements in modern computer and given the limitations of traditional experimental biomechanical testing, finite element analysis presents several advantages in spine biomechanics research. It allows for the elimination of specimen variability and also allows the user to voluntarily control the conditions of the study. Additionally, finite element analysis (FEA) allows for the testing of externally applied forces as well as the analysis of internal stresses of the experimental subject. In the present study, we develop and validate a complete three-dimensional finite element model of DS in order to build the digital platform for further biomechanical study of DS.

## Materials and methods

### Data acquisition

Anterior-posterior and lateral X-rays of the lumbar spine of a 55-year-old female DS patient showed a coronal Cobb angle of 10.8°. The curved segment was from L1 to L5 (including the lower thoracic vertebrae). Magnetic resonance imaging (MRI) revealed spinal deformity and myelodysplasis. Congenital scoliosis and adult idiopathic scoliosis were ruled out, and according to the diagnostic standard of DS (a coronal Cobb angle greater than 10°), the scoliosis was diagnosed as DS. Using a SOMATOM SENSATION 16 spiral CT (Siemens, Munich, Germany), transverse scanning was done on the lumbar segment from T12 to S1 in 0.75-mm slices, yielding 383 two-dimensional CT images of the spinal column. All CT images were saved in DICOM format.

### Building of three-dimensional solid model

**Fig 1:**
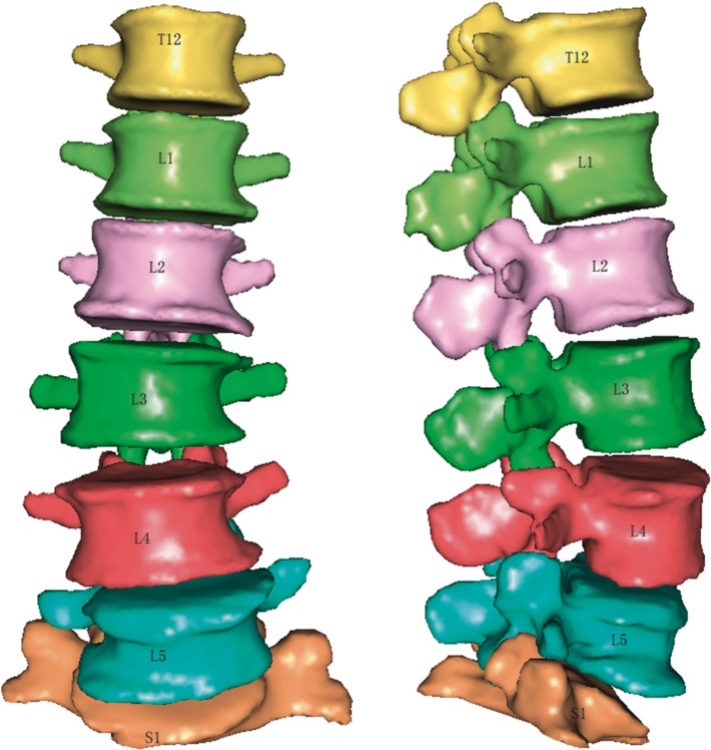
The solid model of spinal column including T12-S1 with the character of coronal Cobb angle about 10° and L4 spondylolisthesis and vertebra osteophyte

The CT images were imported into a finite element modeling system that is specially built for biomechanics research (E-feature Biomedical Modeler, Hui Qing Information Technology, Shanghai, China), hereafter referred to as the “Modeler”. Three-dimensional solid models of each level of the lumbar spine from T12 to S1 were formed from the careful segmentation and reconstruction using the image segmentation and tissue automatic extraction features of the Modeler (Fig. 1).

### Finite element mesh generation of the solid model

The solid model of each vertebra was divided into a high-quality mesh using the self adapting dynamic biomechanical finite element grid of the Modeler. The length of the mesh was designated as 2 mm. The surface elements of each vertebral body were selected (except for the facet joint and intervertebral disc) using submodel design tools of the Modeler and were used to form shell elements with an average thickness of 1.2 mm to represent the cortical bone. The lamina terminalis of the intervertebral disc was simulated with 1-mm shell elements, and the nucleus pulposus was simulated as an incompressible viscoelastic liquid. The annulus fibrosus was modeled as groundmass with collagen fiber buried within it. The groundmass was constituted with three-tier continuous rings, and the collagen fiber was constituted with eight-tier cord elements only bearing tensile stress. The collagen fibers were arranged as scissors in the rings, and the included angle with the surface of intervertebral disc was ±30° on average. Using the graphical interactive modeling tools of the Modeler, the cartilage of each facet joint of each vertebra was swept outwards to form a one-tier 0.6-mm mesh as the cartilage of facet joint, which was simulated as a surface-to-surface contact element with its primary interval of 0.5 mm and with a friction coefficient of 0.1. Using the graphical interactive modeling tools of the Modeler, the anatomical positions of the main ligaments of the lumbar segment were accurately selected, and the ligaments were simulated with cord elements, including the anterior longitudinal ligament, the posterior longitudinal ligament, the supraspinous ligament, the interspinous ligament, the ligamentum flavum, the intertransverse ligament, and the capsular ligament of the facet joint. The texture parameter of each ligament was defined as nonlinear as shown in Table 1 [3, 4].

**Table 1:** Ligament load/deformation properties

Anterior longitudinal ligament		Posterior longitudinal ligament		Supraspinous ligament		Ligamentum flavum		Capsular ligament	
Deformation force		Deformation force		Deformation force		Deformation force		Deformation force	
(mm)	(N)	(mm)	(N)	(mm)	(N)	(mm)	(N)	(mm)	(N)
1.4	12	1.0	9.65	1.3	4.2	1.9	6.7	1.8	6.7
2.7	18	2.0	17.15	2.7	6.1	3.9	11	3.9	11
4.1	22.5	3.0	23.76	4.0	7.4	5.8	13.7	5.8	13.7
5.4	27.15	4.0	28.60	5.4	8.2	7.7	15.7	7.7	15.7
6.8	30	5.0	31.60	6.7	8.8	9.7	16.85	9.7	16.85

### Material properties

DS includes degeneration of the intervertebral disc, facet joints, bone, and ligaments, often with some degree of osteoporosis. Therefore, the properties of each material were specifically chosen to model the behavior of DS. The type and number of elements and material attributes were defined as shown in Table 2 [5, 6].

**Table 2:** Element type and material attributes of DS finite element model

Tissue and area	Element type	Modulus of elasticity (MPa)/Poisson ratio	Thickness cross section area
Cortical bone	6 node points triangular facet shell element	8000/0.3	1.2 mm
Cancellous bone	10 node points tetrahedron solid element	34/0.3	
Lamina terminalis	10 node points tetrahedron solid element	4000/0.4	0.5 mm
Groundmass of annulus fibrosus	10 node points tetrahedron solid element	16/0.4	
Nucleus pulposus	10 node points tetrahedron solid element	8/0.45	
Fiber of annulus fibrosus	2 node points cord element	–	
Articular cartilage	10 node points tetrahedron solid element	1000/0.3	0.5 mm
Contact face of superior articular process	6 node points triangular facet object element		
Contact face of inferior articular process	6 node points triangular facet shell element		
Anterior longitudinal ligament	2 node points cord element		75.9 mm2
Posterior longitudinal ligament	2 node points cord element		51.8 mm2
Ligament umflavum	2 node points cord element		78.7 mm2
Capsular ligament	2 node points cord element		102.5 mm2
Interspinous ligament	2 node points cord element		36.3 mm2
Supraspinous ligament	2 node points cord element		75.7 mm2
Intertransverse ligament	2 node points cord element		40.5 mm2

The three-dimensional finite element model of the whole lumbar spine was imported into ANSYS 12.0 finite element analysis software. All the linear tetrahedrons and shell elements were converted into nonlinear 10-node tetrahedron and 6-node shell elements in order to improve the accuracy of numerical calculation.

### Geometric validation

The finite element model of DS was contrasted to the patient’s X-ray plate to validate the model’s geometric similarity according to their coincidence. The indexes of testing included coronal Cobb angle and lumbar lordosis angle.

### Experimental validation

In accordance with an in vitro biomechanical experiment carried out by Chen et al. [7], the spinal column from L1 to S1 was selected from the finite element model of DS. The model was first repositioned with a slipped L4 vertebral body similar to the aforementioned study by Chen et al. Then, translation and rotation of all nodes of the S1 vertebral body and undersurface of the spinous process were constrained. The surface of the L1 vertebral body was loaded to 10 Nm in 10 substeps in nodal load mode. The model was loaded in flexion, extension, left lateral bending, right lateral bending, left rotation, and right rotation, and the calculated results were compared with the experimental results of the elderly population in the in vitro experiment conducted by Chen et al. To obtain a more effective comparison, the material properties of the normal lumbar spine [5] shown in Table 3 were assigned to the DS model and the same validation as mentioned above was carried out. The calculated motions in each mode of loading were compared with the experimental results of the young people in the Chen biomechanics test.

**Table 3:** Element type and material attributes of normal lumbar finite element model

Tissue and area	Element type	Modulus of elasticity (MPa)/Poisson ratio	Thickness cross-section area
Cortical bone	6 node points triangular facet shell element	12,000/0.3	1.2 mm
Cancellous bone	10 node points tetrahedron solid element	100/0.3	
Lamina terminalis	10 node points tetrahedron solid element	4000/0.4	0.5 mm
Groundmass of annulus fibrosus	10 node points tetrahedron solid element	4.2/0.45	
Nucleus pulposus	10 node points tetrahedron solid element	4/0.49	
Fiber of annulus fibrosus	2 node points cord element	–	
Articular cartilage	10 node points tetrahedron solid element	1000/0.3	0.5 mm
Contact face of superior articular process	6 node points triangular facet object element		
Contact face of inferior articular process	6 node points triangular facet shell element		
Anterior longitudinal ligament	2 node points cord element		75.9 mm^2^
Posterior longitudinal ligament	2 node points cord element		51.8 mm^2^
Ligamentum flavum	2 node points cord element		78.7 mm^2^
Capsular ligament	2 node points cord element		102.5 mm^2^
Interspinous ligament	2 node points cord element		36.3 mm^2^
Supraspinous ligament	2 node points cord element		75.7 mm^2^
Intertransverse ligament	2 node points cord element		40.5 mm^2^

## Results

**Fig. 2:**
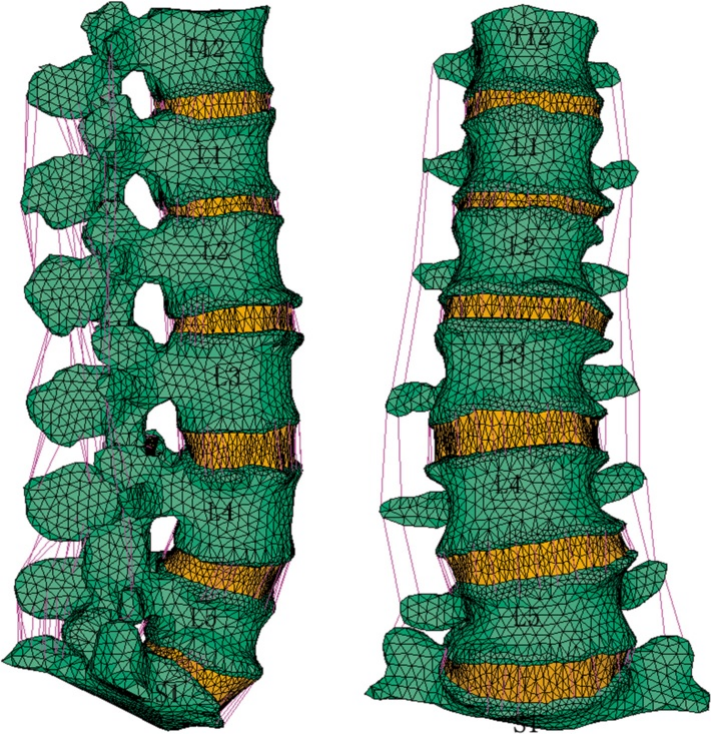
The integral three-dimensional finite element model of DS (with coronal Cobb angle about 10°) including T12 to S1

The integral three-dimensional finite element model of DS including T12 to S1 consisted of 113,682 solid elements, 686 cable elements, 33,329 shell elements, 4968 objective elements, 4968 contact elements, totaling 157,635 elements, and 197,374 node points. The model included geometric nonlinearity, material nonlinearity, and contact nonlinearity. The integral three-dimensional finite element model of DS including T12 to S1 is shown in Fig. 2.

### Geometric validation

**Fig. 3:**
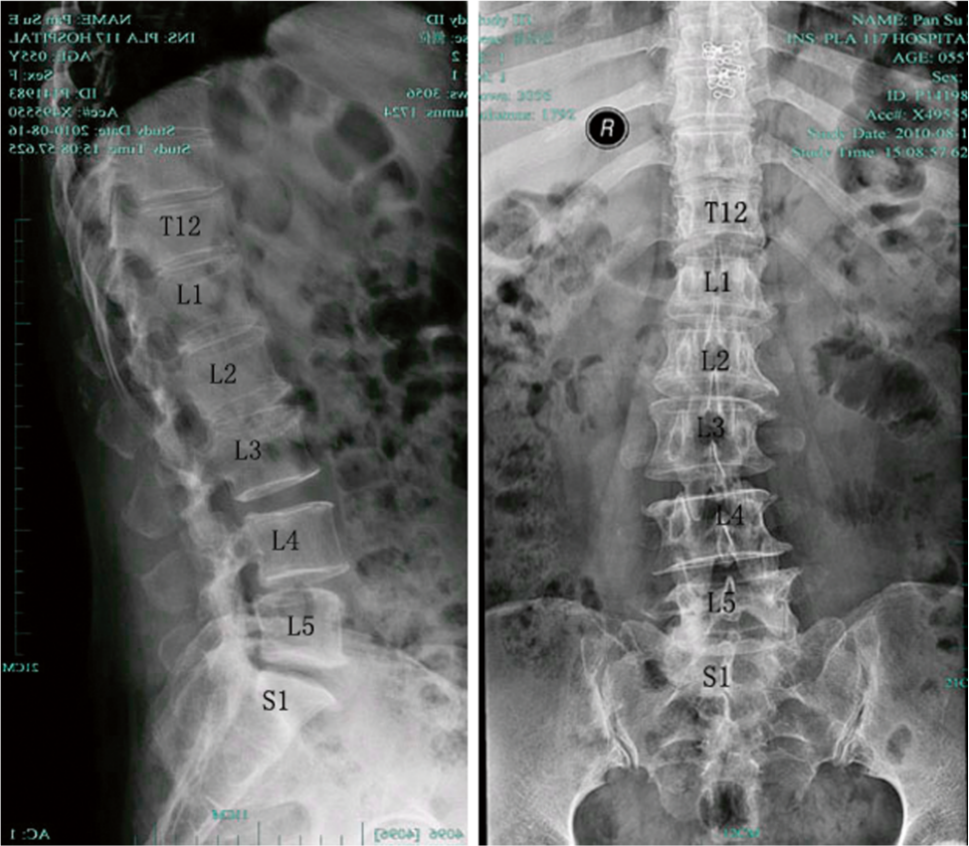
DS patient’s X-ray plate of L-Spine PA and LAT with coronal Cobb angle about 10° and L4 spondylolisthesis and vertebra osteophyte

The model of DS reflected the disease attributes of DS and the geometric features of the patient accurately. The difference of coronal Cobb angle and lumbar lordosis angle was less than 1° as shown in Fig. 3 and Table 4.

**Table 4:** Comparison of finite element model of DS and X-ray

Contents	X-ray	FE model of DS
Alignment of vertebral body	L4° Spondylolisthesis	L4° Spondylolisthesis
Cobb’s angle	10.8°	10.1°
Lordosis angle	24.4°	23.5°

### Experimental validation

The results of the finite element model of DS agreed closely with those obtained from the biomechanical test with an error less that 24 % shown in Table 5.

**Table 5:** Comparison of DS finite element model and in vitro biomechanical spinal mobility (°)

Group mobility	In vitro young group	FE model (normal)	In vitro old group	FE model (degeneration)
Flexion	44.95 ± 8.34	41.34	39.84 ± 6.09	35.41
Extension	23.74 ± 4.63	30.15	21.08 ± 3.85	27.59
Left bending	30.14 ± 5.04	31.42	28.20 ± 5.28	28.01
Right bending		28.81		26.55
Left rotation	23.27 ± 4.10	29.40	21.40 ± 3.46	27.07
Right rotation		31.28		31.86

## Discussion

Spine biomechanics research includes theoretical methods, such as finite element analysis, and experimental methods, such as animal, physical, and cadaveric models. Because of the variability of the spine in both structure and material properties and coupled with the limitations of experimental methods for special or rare cases, FEA has better predictive capability for complex structures and rare situations. The meshing of complex cross-sectional area found in spinal anatomy and the ability to tailor the distribution of nodes for specific geometry makes finite element modeling ideal for understanding DS cases [8]. Compared with cadaveric biomechanical experiments, finite element analysis has some advantages, such as a reduction in the cost of the experiment, shorter experimental time, eliminating the individual variability between specimens and allowing the user to freely control the experimental conditions. It can not only allow for the testing of outside forces but also can quantitatively analyze the changes of internal stress of an experimental subject, which cannot be done in a cadaveric biomechanical experiment [9].

In the 1970s, the finite element technique was introduced to the study of biomedicine and orthopedic biomechanics. In 1976, Andriacchi et al. [10] were the first to apply the method of finite element analysis to the domain of scoliosis by simulating the therapeutic effect of the Milwaukee brace to treat idiopathic scoliosis. With the increasing development of the modern computer, biomechanical research of scoliosis using finite element analysis has become more ubiquitous, but has been mainly limited to idiopathic scoliosis. The biomechanical research of DS is seldom seen with the exception of the finite element model built by Kim [11] studying the stress changes of nerve roots.

Through different imaging modalities, the ability to generate multiple radiographic studies of the same subject facilitates models with comprehensive data sets. The need for comprehensive studies on the same spinal column increases the fidelity of the computational model.

In the present study, a T12 to S1 finite element model of DS was built based on the CT scan of a DS patient. Compared to Kim’s DS model, the one presented here represents a more complete model of the characteristics of DS. Kim’s DS model was built based on the CT scan of a 46-year-old patient without spinal malformation and only included the four vertebrae from L2 to L5. Its scoliosis model was built after redecoration and only included two types: lateral curvature and lateral curvature with rotation. Our finite element model was built on the basis of DS features, which included the degeneration of the intervertebral disc, the facet joint, and the most common comorbidity, osteoporosis. Our DS model reflected the degenerative features in the material properties, making the model more accurate. Moreover, our model was built on the most common type of DS (scoliosis including at least three segments with the coronal Cobb angle less than 20°), which was representative and could provide the platform for the further research of DS biomechanics.

Finite element analysis is a method of computer simulation in which the reference data is usually obtained from traditional experiments. The accuracy of material properties assigned to tissues, the partitioning of meshes, the type of elements used, and the load and boundary conditions directly affect the accuracy of a finite element model. The validation of a finite element model may consist of two stages. The first is the comparison of the appearance and structure of the model with the simulated object. The model presented in this study demonstrated excellent agreement with the structure and appearance of the simulated object. The second stage of validation consists of the comparison of the results obtained from the model with that of a similar experiment or from results obtained from published literature. Because of the difficulty of obtaining cadaveric specimens with DS, in vitro biomechanical experiments of DS cannot be found in the literature, so the second stage of validation was completed with reference to an in vitro biomechanical experiment of lumbar segments. When comparing the results obtained from the normal model and the DS model to the data presented by Chen et al., it is clear that the normal model exhibits behavior similar to that of the young population even though the geometry matches that of a patient with DS. It is also clear from the data presented that the range of motion of the DS model, with material properties defined specifically to mimic the characteristics of DS, is similar to that of the elderly population. This finding underscores the importance of assigning disease-appropriate material properties in the construction of a finite element model of DS.

## Conclusions

According to the validations of geometric similarity to the object of study and in vitro biomechanical experiment of lumbar segments, the three-dimensional finite element model of DS built in this study is reliable and effective and can be used to carry out further biomechanical research of DS. Moreover, the importance of defining appropriate material properties from comprehensive data sets for finite element analysis for such studies has further been elucidated.

## Abbreviations

CT: computed tomography
DS: degenerative scoliosis
FEA: finite element analysis
MRI: magnetic resonance imaging
